# High *in vitro* and *in vivo* synergistic activity between mTORC1 and PLK1 inhibition in adenocarcinoma NSCLC

**DOI:** 10.18632/oncotarget.27930

**Published:** 2021-04-13

**Authors:** Elodie Montaudon, Rania El Botty, Sophie Vacher, Olivier Déas, Adnan Naguez, Sophie Chateau-Joubert, Damien Treguer, Ludmilla de Plater, Leïla Zemoura, Fariba Némati, André Nicolas, Alain Chapelier, Alain Livartowski, Stefano Cairo, Catherine Daniel, Marie Brevet, Elisabetta Marangoni, Didier Meseure, Sergio Roman-Roman, Ivan Bieche, Nicolas Girard, Didier Decaudin

**Affiliations:** ^1^Laboratory of Preclinical Investigation, Department of Translational Research, Institut Curie, PSL University, Paris, France; ^2^Department of Genetics, Institut Curie, Paris, France; ^3^Xentech, Evry, France; ^4^BioPôle Alfort, National Veterinary School of Alfort, Maisons Alfort, France; ^5^Department of Pathology, Hôpital Foch, Suresnes, France; ^6^Department of Tumor Biology, Institut Curie, Paris, France; ^7^Department of Thoracic Surgery, Hôpital Foch, Suresnes, France; ^8^Institut du Thorax Curie Montsouris, Institut Curie, Paris, France; ^9^CYPATH, Centre Léon Bérard, Lyon, France; ^10^Department of Translational Research, Institut Curie, PSL University, Paris, France; ^11^Department of Medical Oncology, Institut Curie, Paris, France; ^*^These authors contributed equally to this work

**Keywords:** NSCLC, Pi3K signalling pathway, mTORC1, RAD001 (everolimus), PLK1

## Abstract

Significant rational is available for specific targeting of PI3K/AKT/mTOR pathway in the treatment of non-small cell lung cancer (NSCLC). However, almost all clinical trials that have evaluated Pi3K pathway-based monotherapies/combinations did not observe an improvement of patient’s outcome. The aim of our study was therefore to define combination of treatment based on the determination of predictive markers of resistance to the mTORC1 inhibitor RAD001/Everolimus. An *in vivo* study showed high efficacy of RAD001 in NSCLC Patient-Derived Xenografts (PDXs). When looking at biomarkers of resistance by RT-PCR study, three genes were found to be highly expressed in resistant tumors, i.e., *PLK1*, *CXCR4*, and *AXL*. We have then focused our study on the combination of RAD001 + Volasertib, a PLK1 inhibitor, and observed a high antitumor activity of the combination in comparison to each monotherapy; similarly, a clear synergistic effect between the two compounds was found in an *in vitro* study. Pharmacodynamics study demonstrated that this synergy was due to (1) tumor vascularization decrease, increase of the HIF1 protein expression and decrease of the intracellular pH, and (2) decrease of the Carbonic Anhydrase 9 (CAIX) protein that could not correct intracellular acidosis. In conclusion, all these preclinical data strongly suggest that the inhibition of mTORC1 and PLK1 proteins may be a promising therapeutic approach for NSCLC patients.

## INTRODUCTION

Specifically targeting the Pi3K signaling pathway in the treatment of non-small cell lung cancers (NSCLC) has now been proposed for more than ten years. Such a therapeutic approach is naturally based, first, on the occurrence of particular Pi3K-related mutations, i.e., *PI3KCA* and *STK11* genes in 6% and 4% of lung adenocarcinomas [1], respectively, and *PI3KCA* and *PTEN* genes in 16% and 8% of squamous cell carcinomas [2], respectively; second, a rational for Pi3K pathway targeting relies on the network existing between this pathway and various and frequent molecular events such as *KRAS* (28%) and *EGFR* (14%) gene mutations observed in adenocarcinomas [1]. However, surprisingly, in the context of tumors defined by a Pi3K activation (*PI3KCA* or *PTEN* mutations), very few clinical trials have tested single-agent Pi3K signaling pathway, all of them showing in fact a modest activity in molecularly unselected NSCLC patients [3], with the mTORC1 inhibitor everolimus (RAD001) [4], and the pan-Pi3K inhibitor Taselisib [5] or Buparlisib [6].

Based on this rationale for the Pi3K pathway targeting in NSCLC, a huge number of clinical trials have consequently tested various Pi3K-based combination of treatments, including chemotherapies such as pemetrexed [7, 8], taxanes [9–13], platinium salts [12–14], EGFR-TKi (afatinib [15], erlotinib [16–18], or gefitinib [19, 20]), radiotherapy [14, 21–23], and others (sunitinib [24] and sunitinib [25]). Tolerability of such combinations was not always acceptable, and, in most situations, conclusions could not be clearly formulated due to the absence of comparison to standard therapies and patient’s randomization. However, almost all of these combinations have been defined according to standard chemotherapies or NSCLC mutations, but not according to specific targets identified as biomarkers of resistance to Pi3K-targeting treatments.

Such an approach, to avoid artificial combinations, may therefore be preferred to circumvent mechanisms of treatment failure and significantly improve the outcome of patients. Our main strategy was therefore, using a panel of NSCLC PDXs, (i) to define predictive markers of response to RAD001 therapy and (ii) to identify possible combinations of treatments that may be able to reverse RAD001 resistance. Hence, using two well-documented Pi3K- and mTORC1-targeted therapies, i.e., BKM120 (Buparlisib) and RAD001 (Everolimus), we have first evaluated *in vivo* responses in NSCLC Patient-Derived Xenografts (PDXs), second defined marker(s) of resistance to these two therapies that inhibition could pharmacologically be proposed, and finally evaluated a new identified and more relevant combination of treatments. We have therefore demonstrated a high synergistic activity between RAD001 and the PLK1 inhibitor volasertib, while exploring mechanisms by which this combination was highly efficient.

## RESULTS

### *In vivo Pi3K* signaling pathway targeted therapy in NSCLC PDXs

Two different therapies have been tested, i.e., the Pi3K inhibitor BKM120 and the mTORC1 inhibitor RAD001 (everolimus), in four PDXs, two of them defined by a *PI3KCA* mutation (ML1 and ML5) and two by a *KRAS* mutation (LCF15 and LCF25). All tested treatments and models are detailed in the Supplementary Table 1.

**Figure 1:**
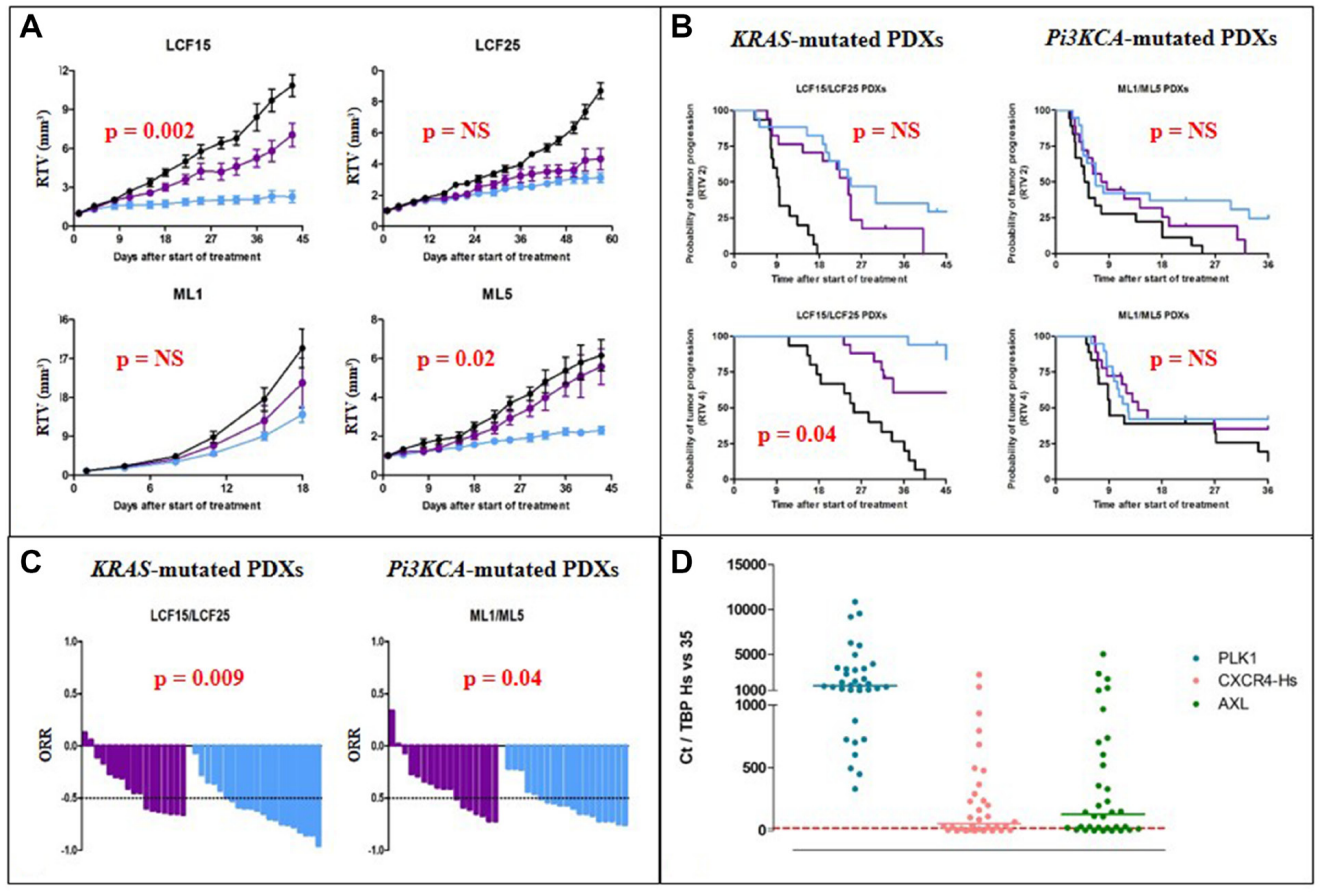
*In vivo* efficacy of Pi3K-targeted therapies in NSCLC PDXs. (**A**) Evolution of the relative tumor volume (RTV) under BKM120 (purple line) and RAD001 (everolimus) (blue line) of the four treated PDXs. (**B**) Probability of progression (doubling time and RTVx4) in *KRAS*- or *PI3KCA*-mutated PDXs after BKM120 (purple line) and RAD001 (everolimus) (blue line) administration. (**C**) Overall response rate in *KRAS*- or *PI3KCA*-mutated PDXs after BKM120 (purple line) and RAD001 (blue line) administration. (**D**) mRNA expression of the human *PLK1*, *CXCR4*, and *AXL* genes in our panel of 34 NSCLC PDXs.

When looking at the Relative Tumor Volume (RTV), we have observed a high and significantly better efficacy of RAD001 in comparison to BKM120 in two models (LCF15 and ML5), and no significant difference in the two other PDXs (LCF25 and ML1) (Figure 1A) (unpaired t test). Hence, through the determination of the probability of progression (two or four-fold RTV increase) (Figure 1B) and of the overall response rate (ORR) (Figure 1C) that allow a merged analysis of *in vivo* experiments, we have observed a significantly higher efficacy of RAD001 in comparison to BKM120 in 2 among the 4 tested PDXs (one KRAS- and one Pi3KCA-mutated models), without differences between *PI3KCA*- or *KRAS*-mutated PDXs; indeed, an ORR lower than -0.5 was observed after BKM120 and RAD001 in 41% and 76% of *KRAS*-mutated-bearing mice, respectively (*p* = 0.009), and in 41% and 74% of Pi3KCA-mutated-bearing mice, respectively (*p* = 0.04). As shown in the Supplementary Table 1, four other PDXs have been treated by BKM120 (LCF4, LCF9, LCF12, and LCF29) and one other model by RAD001 (LCF29); overall, the median time of the RTVx2-probability of progression was 16 and 22 days for BKM120 and RAD001, respectively (Supplementary Figure 1).

Moreover, two different combinations have been tested: First, in one PDX defined by both *PI3KCA* and *EGFR* mutations, i.e., the LCF12 PDX, we have evaluated the efficacy of BKM120 + one EGFR-targeted therapy (cetuximab, erlotinib, or afatinib); we have observed no significant improvement of antitumor response after the three combinations in comparison to the most efficient monotherapy (Supplementary Figure 2), with a significant higher efficacy of afatinib in comparison to cetuximab and erlotinib (*p* < 10^-2^). Second, we have combined in three models RAD001 and the MEK1/2 inhibitor selumetinib, and we did not observe a significant benefit of the combination in comparison to each monotherapy, whatever the mutational status of tested PDXs (*KRAS*-mutated/*PI3KCA*-wt LCF15 and LCF25 PDXs; *KRAS*-wt/*PI3KCA*-mutated ML5 PDX) (Supplementary Figure 3).

Overall, our first *in vivo* results showed a high efficacy of RAD001 in NSCLC PDXs, independently of the *PI3KCA* mutational status. Hence, the next step of our work was to define predictive markers of response to RAD001.

### Determination of predictive markers of response to RAD001 (everolimus) in NSCLC PDXs

In order to define predictive markers of response to RAD001, we used real-time quantitative RT-PCR assays to quantify the mRNA expression of a large number of selected genes. We included a number of genes known to be involved in various cellular and molecular mechanisms associated with tumorigenesis, and altered (mainly at the transcriptional level) in various cancers. These genes encode proteins involved in cell cycle control, cell-cell interactions, signal transduction pathways, apoptosis, angiogenesis, etc. We more particularly focused on the expression of genes related to the Pi3K and MAPK signaling pathways. Basal expression of this panel of candidate genes have been evaluated and correlated to the individual *in vivo* response (a tumor was considered as a responding tumor when its individual response rate was lower to -0.5). The list of studied genes is presented in the Supplementary Table 2, as well as the results of the statistical analysis.

When looking at predictive markers of response to BKM120, only three genes for which high expression was predictive of response to therapy have been identified, i.e., *AREG*, *RASSF1*, and *ALDH1A1* genes. In contrast, a higher number of predictive genes have been identified as predictive markers of response to RAD001. Among these genes, eleven genes were significantly highly expressed in responding tumors (genes have been indicated in red) and eleven were significantly highly expressed in resistant tumors (genes have been indicated in green); among these last genes (in green), only three encode for a protein that could be inhibited by a specific targeted therapy, namely *PLK1*, *CXCR4*, and *AXL* (Supplementary Table 2).

Focusing on these three genes, we have then evaluated basal expression in our NSCLC PDXs panel (34 models). We therefore observed that the median of mRNA level values (as determines in the “Material and Methods” section) was 1526, 55, and 131 for *PLK1*, *CXCR4*, and *AXL* genes, respectively (Figure 1D). We thus examined the prognostic value of expression of these three genes in a publicly available lung cancer database (KMPLOT; http://kmplot.com) [26]. This database contains gene expression data and overall survival (OS) information for 3452 lung cancer patients (all histology, as well as adenocarcinoma and squamous cell carcinoma). We have therefore found that only a high gene expression of *PLK1* has a pejorative impact on the OS of adenocarcinoma but not squamous cell carcinoma (Supplementary Figure 4).

Considering the fact that (i) *PLK1* was the only one of these three identified genes that expression was high in our NSCLC PDXs, (ii) high *PLK*1 gene expression was the only one of the three tested genes having a pejorative impact on NSCLC patients’ OS, and in order to reverse RAD001 resistance, we then evaluated the *in vivo* efficacy of only one combination of treatments, i.e., RAD001 plus the PLK1 inhibitor volasertib.

### *In vivo* efficacy of RAD001 (everolimus) + volasertib in NSCLC PDXs

**Figure 2:**
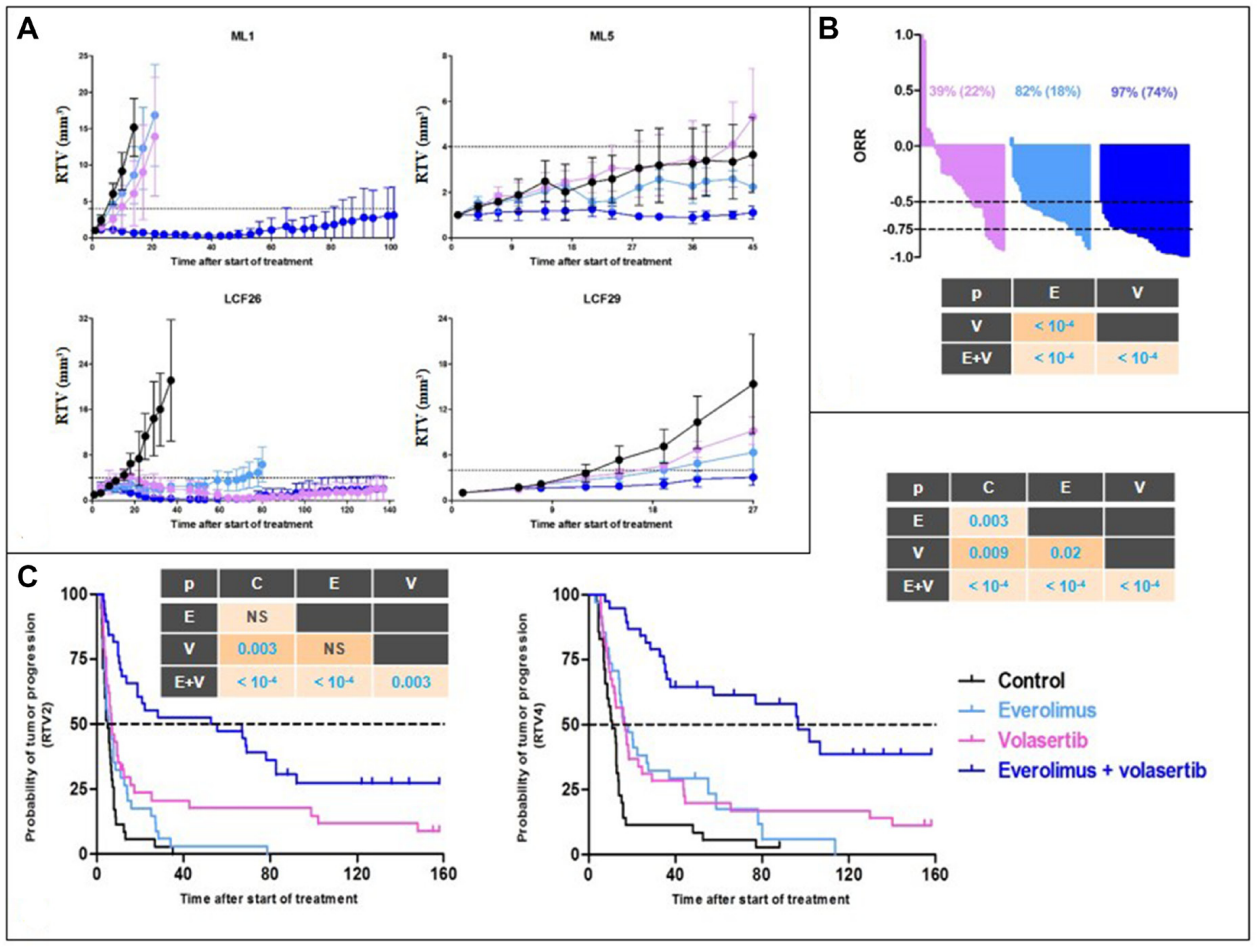
*In vivo* efficacy of RAD001 (everolimus) + volasertib in NSCLC PDXs. (**A**) Evolution of the relative tumor volume (RTV) under RAD001 (blue line), volasertib (purple line), and combination (dark blue line) of the four treated PDXs. (**B**) Overall response rate in all treated PDXs after RAD001 (blue line), volasertib (purple line), and combination (dark blue line). (**C**) Probability of progression (doubling time and RTVx4) in in all treated PDXs after RAD001 (blue line), volasertib (purple line), and combination (dark blue line).

Four PDXs have been used for this *in vivo* part of the study, i.e., LCF26, LCF29, ML1, and ML5 (the main histopathological and mutational features of these models are presented in the Supplementary Table 3). In all tested PDXs, except LCF29, we have observed a significant, but variable, improvement of the antitumor efficacy of RAD001 + volasertib in comparison to each monotherapy (Figure 2A). Similarly, when the data of the four treated models have been merged, the overall response rate lower to -0.5 (and -0.75) was 82% (18%), 39% (22%), and 97% (74%) after RAD001, volasertib, and RAD001 + volasertib, respectively (*p* < 10^-4^) (Figure 2B). Finally, the median time of the RTVx4-probability of progression was 18, 17, and 96 days after RAD001, volasertib, and RAD001 + volasertib, respectively (*p* < 10^-4^) (Figure 2C). We have finally treated a squamous NSCLC PDX, i.e., SC131, and we did not observe higher efficacy of RAD001 + volasertib in comparison to RAD001 alone (Supplementary Figure 5).

Considering the dramatic efficacy of RAD001 and volasertib combination, we therefore focused our study to define mechanism of such an activity.

### Pharmacodynamics study of RAD001 (everolimus) + volasertib combination

**Figure 3:**
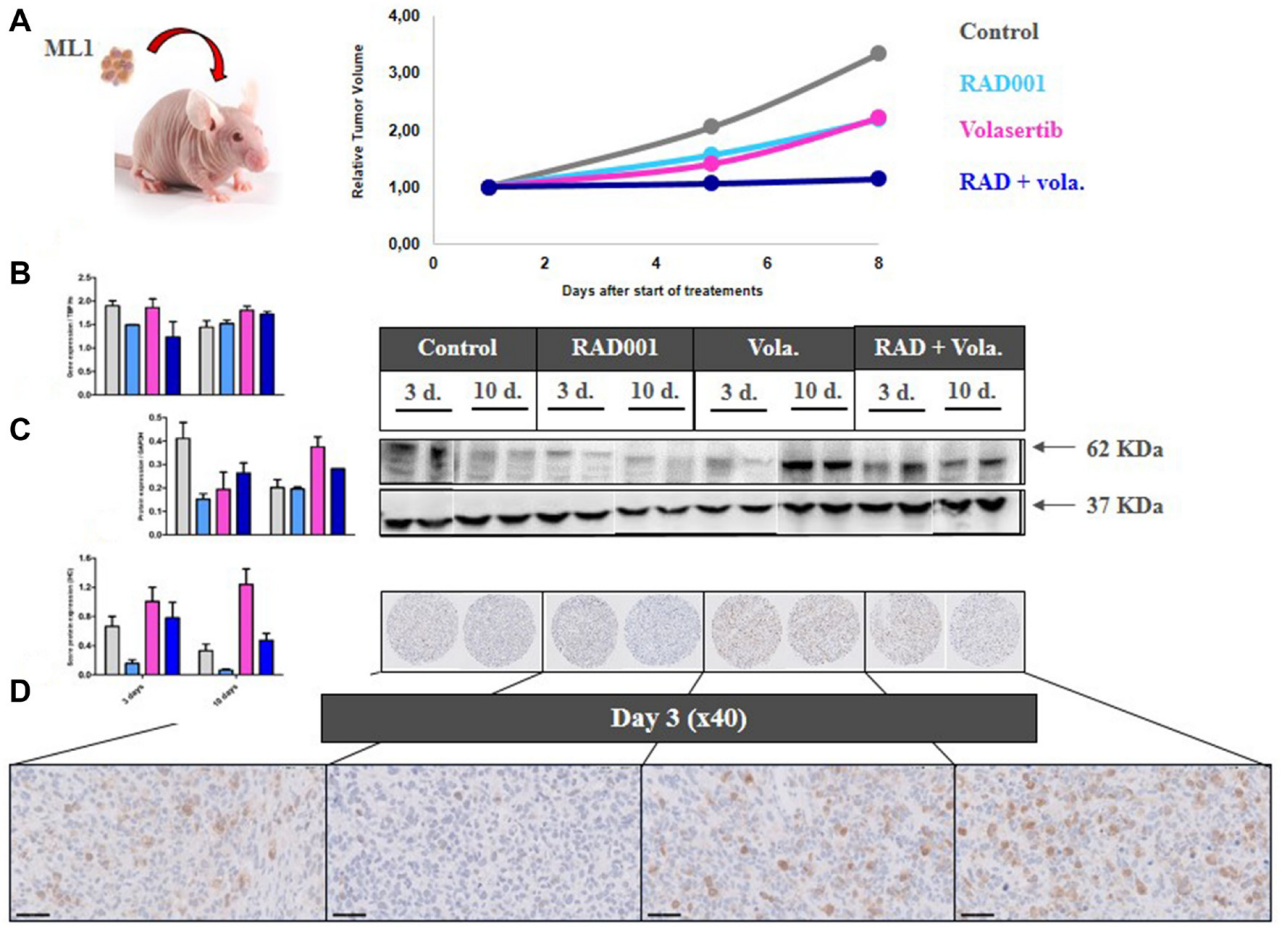
PD study in ML1 PDX and PLK1 expression. (**A**) *In vivo* schedule of the Pharmacodynamics (PD) study. Tumors have been collected at days 3 and 10 after start of treatment. (**B**) RT-PCR study of *PLK1* gene expression. (**C**) Western Blot study of PLK1 protein expression. (**D**) IHC study of PLK1 protein expression.

To perform a relevant PD study, we grafted the ML1 PDX in which a high effect of the combination of treatments was observed, and we collected tumors at two times after start of treatments, i.e., 3 and 10 days (Figure 3A). Moreover, various methodologies have been used, namely RT-PCR gene expression, Western Blot, and/or Immunohistochemistry protein expression.

We first evaluated the expression under therapies of various cell cycle-involved proteins. Hence, we observed that volasertib, particularly at day 10, induced a significant increase of PLK1 expression at RNA and protein levels (Figure 3B–3D). RNA (using RT-PCR) and protein (using IHC) Ki67 expression was not affected by any treatments (Supplementary Figure 6A and 6B).

**Figure 4:**
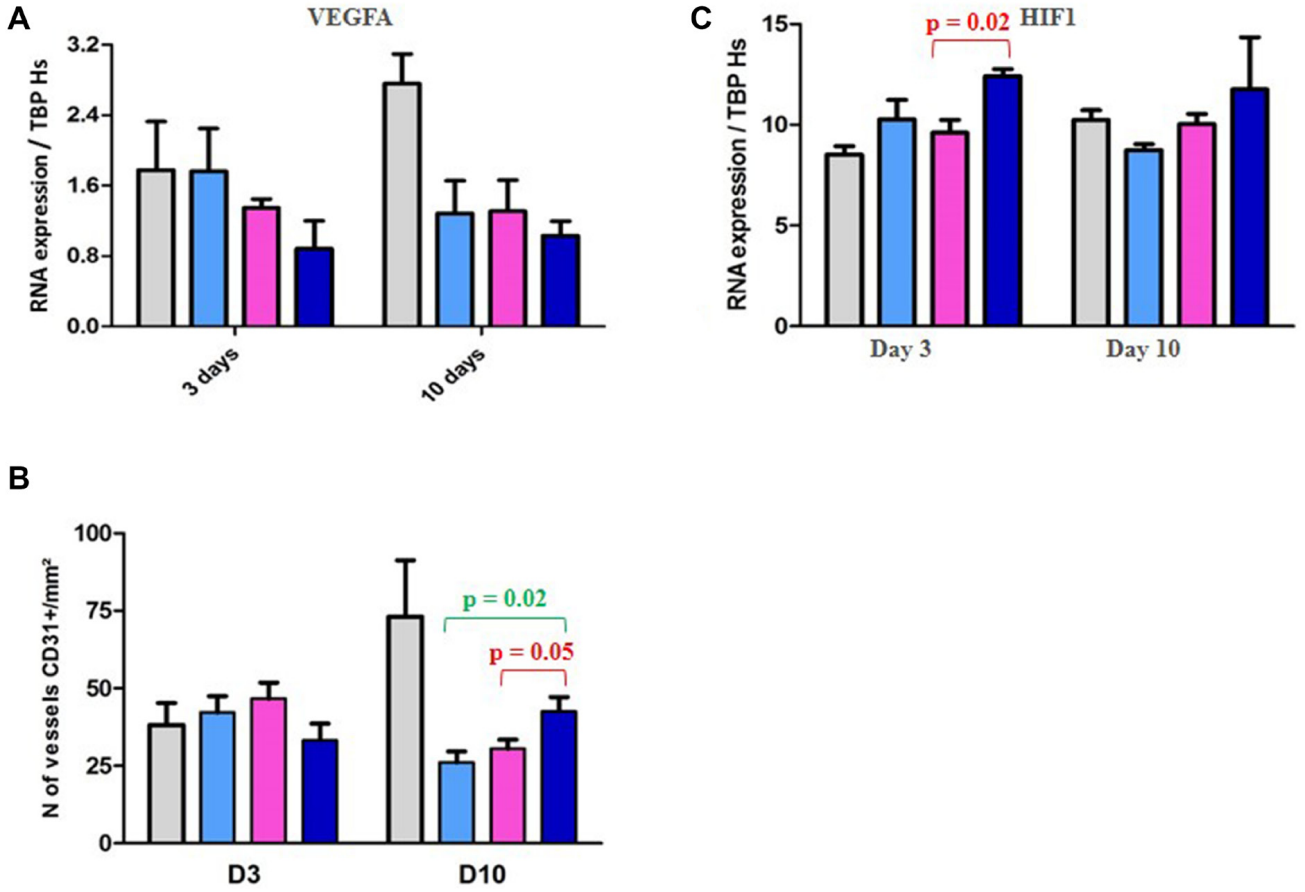
PD study in ML1 PDX and vascularization/HIF1 expression. (**A**) Figure RT-PCR study of VEGFA gene expression. (**B**) IHC study of the number of vessels CD31+/mm². (**C**) PD study in ML1 PDX and HIF1 gene expression. In the Figure 4A, 4B and 4C, colored bars correspond to control (grey), RAD001 (blue), volasertib (pink), and combination (dark blue).

**Figure 5:**
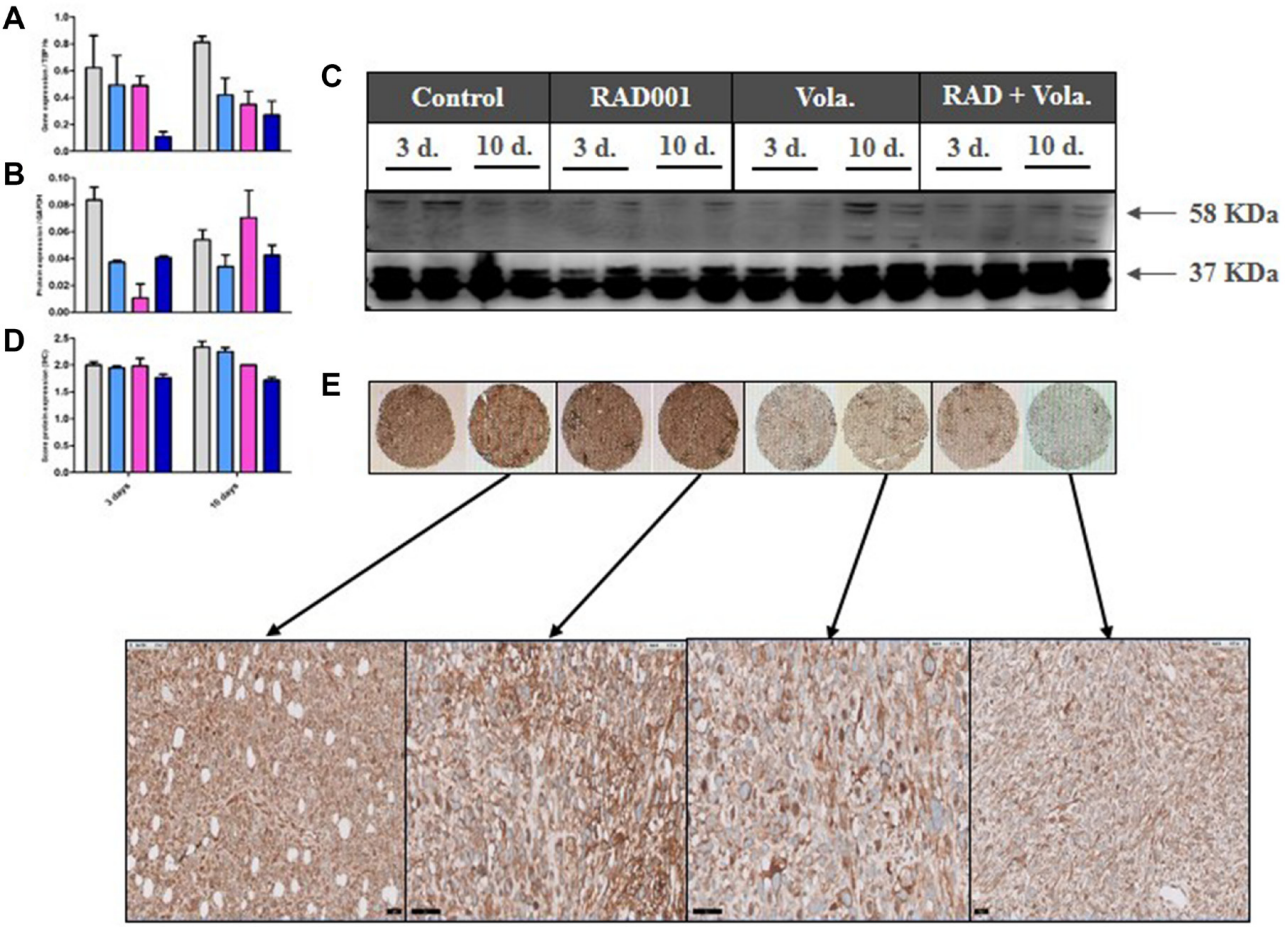
PD study in ML1 PDX and CAIX expression. (**A**) RT-PCR study of CA9 gene expression. (**B**, **C**) Western Blot study of CAIX protein expression. (**D**, **E**) IHC study of CAIX protein expression. In the Figure 4A, 4B and 4D, colored bars correspond to control (grey), RAD001 (blue), volasertib (pink), and combination (dark blue).

In a second time, we have evaluated the outcome of vascularization under therapies. We have first evaluated the outcome of vascularization under therapies. We have shown a drop of the *VEGFA* gene expression, as well as of the number of CD31+ vessels per mm² (Figure 4A and 4B), in the three treated groups of tumors (RAD001 alone, volasertib alone, and RAD001 + volasertib); particularly, the number of vessels was significantly lower in the combination group than each monotherapies. Consequently, probably due to hypoxia-induced treatments, we have observed an increase of *HIF1* gene expression under therapies, preferentially after RAD001 + volasertib combination in comparison to the volasertib group (*p* = 0.02) (Figure 4C). Finally, we have also evaluated Carbonic anhydrase IX expression, showing a dramatic drop of both *CA9* gene and Carbonic Anhydrase 9 (CAIX) protein expression in all tested treatments, but with a maximal impact after RAD001 + volasertib combination (Figure 5).

Finally, we have studied the impact of all tested treatments in both Pi3K and MAPK signaling pathways. As expected, we have shown a high decrease of S6 phosphorylation after RAD001 administration alone or in combination (Supplementary Figure 7A and 7B), but not volasertib treatment. In contrast, we have observed that volasertib significantly increased phosphorylation of MEK1/2 and ERK, particularly when combined with RAD001 (Supplementary Figure 8A and 8B).

All these PD results therefore suggest that, through vascularization alteration-dependent hypoxia, RAD001 + volasertib combination induced an increase of HIF1 and a decrease of CAIX proteins. To validate such a hypothesis, we then performed an *in vitro* study.

*In vitro* **study of RAD001 (everolimus) + volasertib combination**

**Figure 6:**
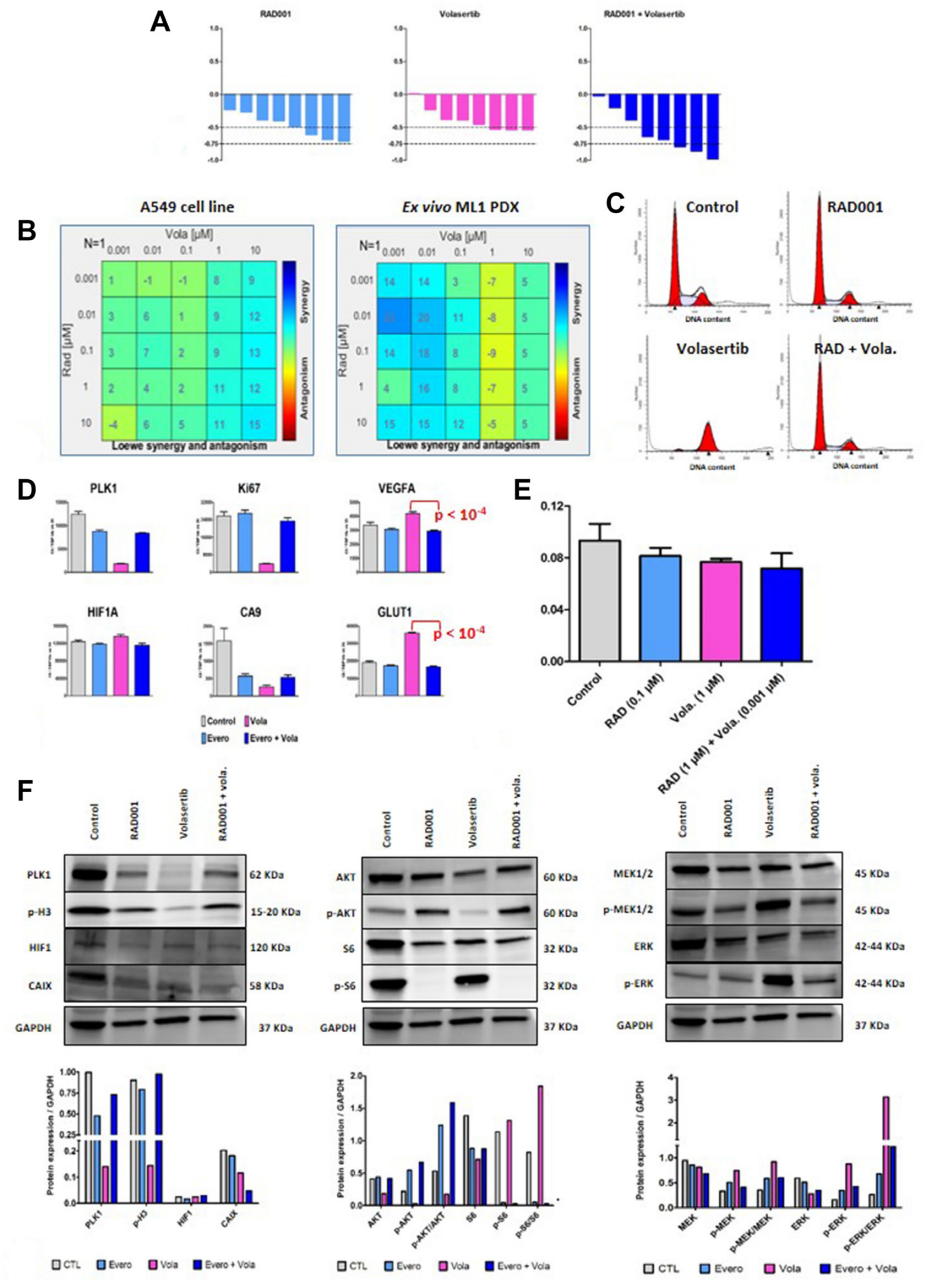
*In vitro* study of RAD001 (everolimus) + volasertib combination. (**A**) *In vivo* efficacy of RAD001 + volasertib in the A549 cell line. The indicated percentage correspond to an overall response rate lower than -0.5 and 0.75 (in bracket). (**B**) Synergy determination between RAD001 and volasertib in the A549 cell line and the *ex vivo* ML1 PDX; cell viability was measured after three days of drug treatment using the MTT assay (Sigma); various drug concentrations, indicated in the Figure, have been used. (**C**) Flow cytometric analyses in the A549 cell line; analyses were performed after twenty-four hours of drug treatment (0.1 μM RAD001, 1 μM volasertib). (**D**) RT-PCR study in the A549 cell line; cells were cultures for three days with tested treatments (0.1 μM RAD001, 1 μM volasertib). Results are expressed as N-fold differences in target gene expression relative to the TBP gene. (**E**) Intracellular pH study in the A549 cell line; analyses were performed after twenty-four hours of drug treatment (0.1 μM RAD001, 1 μM volasertib). (**F**) Western Blot study in the A549 cell line; cells were cultures for three days with tested treatments (0.1 μM RAD001, 1 μM volasertib).

In order to perform *in vitro* study using the A549 cell line, we have performed an *in vivo* experiment and have confirmed the additive but not significantly effect of RAD001 + volasertib in comparison to each monotherapy (Figure 6A).

We then performed various *in vitro* analyses. First, RAD001 + volasertib combination was assessed for synergy based on cell viability and according to the Loewe independence model. *In vitro* experiments were done using the A549 adenocarcinoma cell line of NSCLC, and the ML1 PDX used in an *ex vivo* assay. As shown in the Figure 6B, we have observed in both models a synergistic activity between RAD001 and volasertib. Second, flow cytometric analyses showed that volasertib induced a cell cycle arrest into the G2 phase, that effect which was not observed after RAD001 + volasertib treatment (Figure 6C and Supplementary Table 4). This result suggest that the synergistic effect between RAD001 and volasertib was not totally dependent to the cell cycle activity of the PLK1 inhibitor. Third, using the A549 cell line, we performed complementary PD study. In this model, we have confirmed that volasertib induced a slight increase of *HIF1* gene expression and a dramatic drop of *CA9* gene expression under therapies (Figure 6D); particularly, we have also observed a highly significant decrease of both VEGFA and GLUT-1 mRNA expression in the combination group in comparison to the volasertib group (Figure 6D). Moreover, we have also observed a highly significant decrease of CAIX protein expression using WB analysis (Figure 6F). Finally, we have shown a slight but significant decrease of the intracellular pH (Figure 6E). Finally, WB study showed an activation of both Pi3K and MAPK pathway after RAD001 + volasertib combination (Figure 6F).

**Figure 7:**
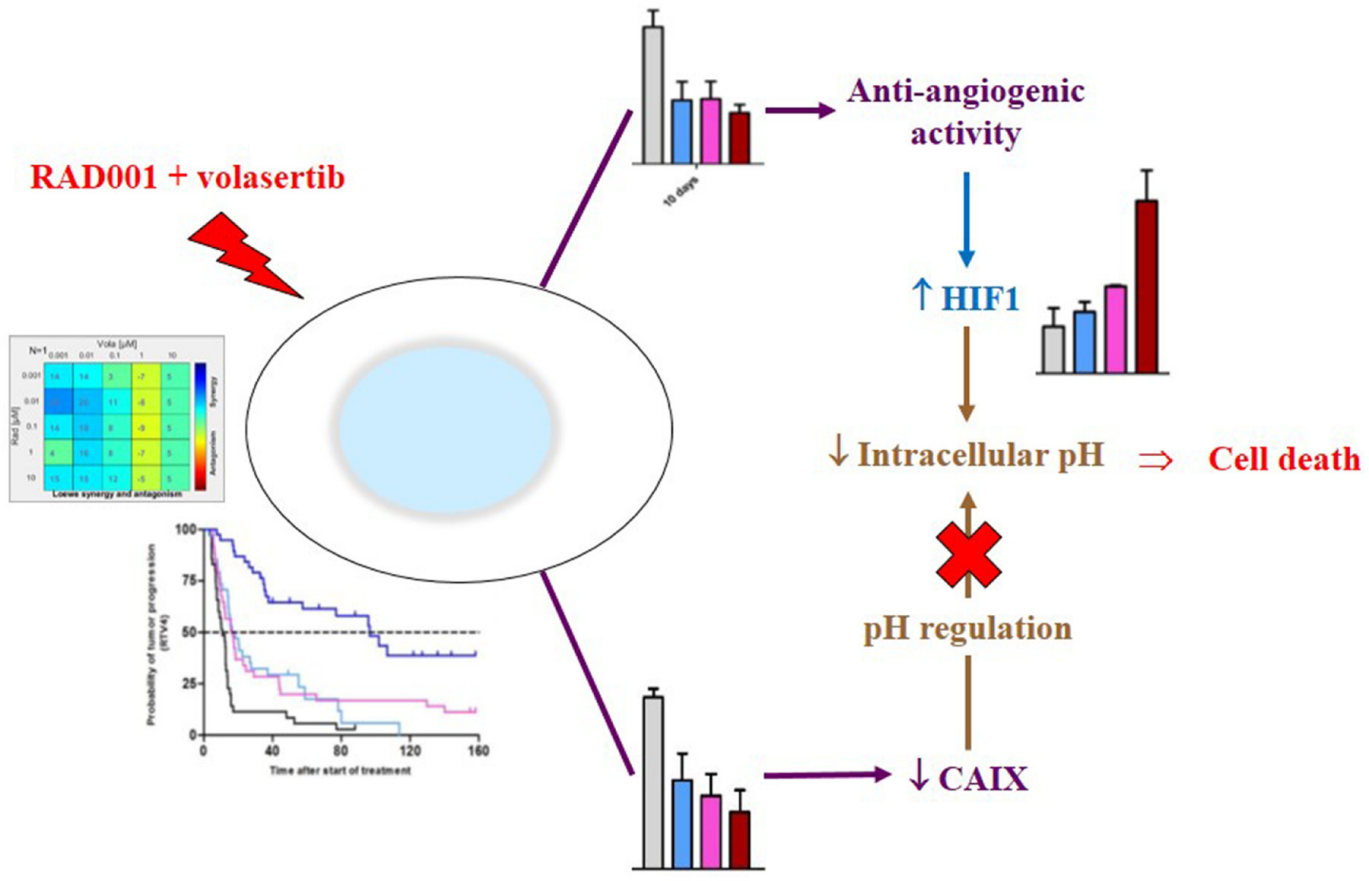
Mechanism of the antitumor activity of the RAD001 (everolimus) + volasertib combination.

In conclusion, considering all of our results, we can formulate the following hypothesis on the additive effect of RAD001 + volasertib in adenocarcinoma NSCLC PDXs (Figure 7): the combination of RAD001 + volasertib induces, first, an antiangiogenic effect that may result to increase HIF1 expression; this increase of HIF1 expression may induce a decrease of the intracellular pH; in parallel, the combination induces a drop of CAIX protein that is not able to regulate anymore intracellular pH. Consequently, the intracellular acidosis could not be circumvent and it induces tumor cell death.

## DISCUSSION

In our study, we have demonstrated that the inhibition of both mTORC1 and PLK1 proteins induced a high and synergistic antitumor activity in various NSCLC PDXs. Such an effect of the combined treatment is due to a double consequence on tumor cells, first through an impact on tumor vascularization with hypoxia, increase of HIF-1α protein expression and intracellular acidosis, and, second, decrease of CAIX protein which is not available to correct acidosis.

Our results are concordant with the fact that the Pi3K signaling pathway may impact the expression of HIF1, VEGF, and CAIX proteins. However, all of these data are not totally unequivocal in the literature: indeed, a first study has shown that Pi3K activation induces HIF1 expression but decreases CAIX expression [27]. Similarly, a second study has shown that HIF1 promotes chemo-resistance by induction of CAIX expression [28]. In contrast, and more recently, a third study has observed the fact that targeting carbonic anhydrase IX may have an antitumor activity in lung cancer [29] or improve antitumor activity of mTOR inhibitors [30]. And similarly, a fourth study has shown an inverse correlation between HIF1 and CAIX expression [31]. Finally, a high CAIX protein expression has largely been considered as having a pejorative prognostic impact in NSCLC [32–34]. Our results, performed in both PDXs and an *in vitro* NSCLC cell line, have clearly shown, in parallel to a strong synergistic activity of RAD001 + volasertib, an increase of HIF1 and a drop of *CA9* gene and CAIX protein expression. Hence, we consider that such a reproducible observation supports its validation and its role in the mechanism of action of the treatment combination.

Polo-like kinase 1 (PLK1), which plays a role in cell-cycle regulation [35], has also been shown to be involved in resistance to standard chemotherapies, such as doxorubicin, paclitaxel, gemcitabine, and cytarabine. In monotherapy, a slight antitumor efficacy was observed, suggesting the fact that PLK1 inhibition may be more promising in combinations of treatments. Such one combination with an inhibitor of PDGF, VEGF, and bFGF receptors has been evaluated in cancer patients, with a disease control in 18/30 evaluable patients in which one partial response in NSCLC [36]. In this context, our study therefore proposes another PLK1 inhibition-based combination which preclinical efficacy appears highly promising. Future clinical trials that assess the combination of PLK1 inhibitors with antiangiogenic agents already part of the standard-of-care in advanced NSCLC, such as bevacizumab or ramucirumab, or next-generation drugs, including multikinase inhibitors such as lenvatinib, would fit with the current unmet need of effective treatment strategies after the failure of platin-based chemotherapy and immune checkpoint inhibitors, possibly integrating assessment of tissue-based biomarkers including PLK1 expression.

Our determination of relevant Pi3K-based therapeutic combination(s) was not supported, by the presence of actual molecular abnormalities, nor by physician therapeutic practices, but by the identification of predictive markers of resistance to Pi3K-based monotherapies. The step-by-step methodology presented here highlights the useful of appropriate preclinical models of human cancers, such as PDXs; this methodology could be summarized, as follow: (1) assessment and validation of an antitumor activity of a treatment X, (2) determination of predictive marker(s) of resistance to the compound X, (3) identification of a second treatment Y able to reverse the dismal impact of a previously defined predictive marker of resistance, and validation of its clinical relevance in terms of its prognostic impact, (4) assessment and validation of an antitumor activity of the combination of the treatments X + Y, (5) comprehensiveness, as far as possible, of the mechanisms of action of this new treatment combination, and, finally, (6) clinical assessment in cancer patients. This methodology may promote more relevant clinical trials and avoid non-efficient combinations, inacceptable toxicities, and expensive and time-consuming studies.

## MATERIALS AND METHODS

### NSCLC PDXs and *in vivo* treatments

Eight NSCLC PDXs have been included in the first *in vivo* pharmacological study. The main histopathological and molecular features of these models are summarized in Supplementary Table 1. PDX models are available from the corresponding author or, for some ones, *via* the EuroPDX Data Portal (https://dataportal.europdx.eu) and the PDXFinder (https://www.pdxfinder.org). Tested treatments were based on strategies tested in the clinic and included: Pi3K signaling pathway targeting with the Pi3K inhibitor BKM120 (oral administration at a dose of 20 mg/kg, 5 days a week) and the mTORC1 inhibitor RAD001 (oral administration at a dose of 2 mg/kg, 5 days a week); EGFR targeting with cetuximab (IP injection at a dose of 25 mg/kg, twice a week), erlotinib (oral administration at a dose of 30 mg/kg, 5 days a week), or afatinib (oral administration at a dose of 20 mg/kg, 5 days a week); and KRAS targeting with the MEK1/2 inhibitor selumetinib (twice oral administration at a daily dose of 50 mg/kg, 5 days a week).

In the second *in vivo* part of the study, five PDXs have been included that main histopathological and molecular features are presented in the Supplementary Table 2. Two treatments have been used as monotherapy or combination: RAD001 which was administered as described above; and the PLK1 inhibitor volasertib (oral administration at a dose of 10 mg/kg, 5 days a week).

### *In vivo* tumor growth and antitumor efficacy

For *in vivo* therapeutic studies, female *Nude* mice (Charles River, France) were xenografted with a tumor fragment of 20–40 mm^3^. Mice bearing growing tumors with a volume of 60–150 mm^3^ were randomly assigned to the control or treatment groups. Between 6 and 10 animals per group have been treated in all experiments. Treatments were started on day 1. Mice were weighed and tumors measured twice a week. Xenografted mice were sacrificed when their tumor reached a volume of 2500 mm^3^. In each *in vivo* experiment, between three to five frozen and formol-fixed tumor tissues have been collected at the time of ethical sacrifice in all treated groups for a further pharmacodynamics (PD) study.

Tumor growth was evaluated by measurement twice a week of two perpendicular diameters of tumors with a caliper. Individual tumor volume, relative tumor volume (RTV), and tumor growth inhibition (TGI) were calculated according to standard methodology [37]. Moreover, to evaluate the overall response rate (ORR) to treatments observed in all treated models according to individual mouse variability, we decided to consider each mouse as one tumor-bearing entity. Hence, in all *in vivo* experiments, a relative tumor volume variation (RTVV) of each treated mouse was calculated from the following formula: [(RTVt/mRTVc)], where RTVt is the relative tumor volume of the treated mouse and mRTVc the median relative tumor volume of the corresponding control group at a time corresponding to the end of treatment. Then, for each treated mouse, we calculated [(RTVV)-1]. Finally, to clearly define the impact of treatments on the tumor progression, we have also evaluated the probability of progression (doubling time and time for RTV x 4), as described [38].

Statistical significance of differences observed between the individual RTVs corresponding to the treated mice and control groups was calculated using the two tailed Mann Whitney test. Statistical significance of ORR between tested treatments was determined using a χ^2^ test. Statistical significance of probability of progression between tested treatments was determined using a student *t* test.

Animal care and use for this study were performed in accordance with the recommendations of the European Community (2010/63/UE) for the care and use of laboratory animals. Experimental procedures were specifically approved by the ethics committee of the Institut Curie CEEA-IC #118 (Authorization APAFiS# 25870-2020060410487032-v1 given by National Authority) in compliance with the international guidelines.

### RT-PCR study

Quantitative values were obtained from the cycle number (Ct value) at which the increase in the fluorescence signal associated with exponential growth of PCR products started to be detected by the laser detector of the ABI Prism 7900 Sequence Detection System (Perkin-Elmer Applied Biosystems, Foster City, CA), using PE Biosystems analysis software according to the manufacturer’s manuals. The *TBP* gene (Genbank accession NM_003194) encoding the TATA box-binding protein (a component of the DNA-binding protein complex TFIID) was quantified as an endogenous RNA control, and each sample was normalized on the basis of its *TBP* content [39]). Results, expressed as N-fold differences in target gene expression relative to the TBP gene and termed “Ntarget”, were determined as Ntarget = 2^ΔCtsample^, where the ΔCt value of the sample was determined by subtracting the average Ct value of the target gene from the average Ct value of the *TBP* gene. The smallest amount of mRNA that was detectible (ΔCt = 35) was used as a reference (basal mRNA level = 1) to normalize the data for xenograft samples. Primers’ sequences are available on request. The conditions of cDNA synthesis and PCR have been described previously [39].

### Bio-analysis *in silico*

KM Plotter data were obtained using the current release of Kaplan Meier Plotter (http://www.kmplot.com) [40], interrogating the database using Affymetrix ID “205880_at” for distant metastasis-free survival (no follow-up threshold). The best cutoff value was automatically selected and biased arrays were excluded from the analysis.

### Western blot study

Proteins were extracted from tumors using Laemmli buffer (50 mM Tris HCL pH 8, 2 mM DTT, 2% SDS, 5% glycerol), supplemented with protease and phosphatase inhibitors. Lysates were resolved on 10% agarose gels, transferred onto nitrocellulose membranes (Bio-Rad, Hercules, CA, USA) and immunoblotted with rabbit antibodies against AKT, p-AKT (ser 473), S6, p-S6 (Ser235/236), ERK, p-ERK (Thr202/Tyr204), MEK, p-MEK (Ser217/221), PLK1, CAIX, HIF1 and GAPDH (Cell Signaling^®^) (Supplementary Table 5). Densitometry readings/intensity ratio of each band have been indicated (Supplementary Table 6). After washes, membranes were incubated with the appropriate horseradish peroxidase-conjugated affinity-purified goat anti-rabbit secondary antibodies (Jackson ImmunoResearch Laboratories, Inc., Interchim). Protein quantification was performed with Image Lab software and normalized on GAPDH expression and means were compared with a Mann-Whitney test. *p-value*s were considered statistically significant for ^*^ *p* < 0.05, ^**^ *p* < 0.01 and ^***^ *p* < 0.001.

### Histopathological study

Immunohistochemistry was performed by using S6, PLK1, CD34, Akt, P-Akt, ERK, P-ERK, HIF-1, MEK, P-MEK antibodies in NSCLC PDXs (Supplementary Table 7). Paraffin-embedded tissue blocks, obtained at the time of the initial diagnosis, were retrieved from the archives of the Department of Biopathology, Institut Curie. Sections of 3 μm were cut with a microtome from the paraffin-embedded tissue blocks of normal breast tissue, preinvasive lesions, and IBCs. Tissue sections were deparaffinized and rehydrated through a series of xylene and ethanol washes. Briefly, key figures included: (i) antigen retrieval in 0.1 mol/L citrate buffer, pH 6or ph9 (Biocare) in a pressure cooker (10 to 20 minutes); (ii) blocking of endogenous peroxidase activity by immersing sections in 3% hydrogen peroxide in methanol for 15 minutes and subsequently rinsing them in water and PBS; (iii) incubation with primary antibodies against the targeted antigen; (iv) immunodetection with a biotin-conjugated secondary antibody formulation that recognizes rabbit and mouse immunoglobulins, followed by peroxidase-labeled streptavidin and linking with a rabbit biotinylated antibody against mouse immunoglobulin G (DAKO SA); and (v) chromogenic revelation with AEC and counterstaining with Mayer’s hematoxylin. All immunostaining was processed by using DAKO automated immunostaining and LEICA BOND RX devices. The specificity of the antibodies was confirmed by doing IHC studies with the same protocol on paraffin-embedded human tissue sections containing lymphocytes. A semiquantitative histologic score (H-score = intensity × frequency) was performed (score 0 = negative staining, score 1 = weak staining, score 2 = moderate staining, score 3 = strong staining).

### *In vitro* cell viability assay

*In vitro* cell viability assays were performed as described by Carita *et al.* [41]. Briefly, cells were seeded in three 96-well plates following a 6 × 6 matrix design. The day after, each drug was added following a matrix dilution format. 1:10 serial dilutions were tested to result in a total of six serial dilutions, including the DMSO control. Cell viability was measured after three days of drug treatment using the MTT assay (Sigma). Results were read using a spectrophotometer, and expressed as relative percentages of metabolically inactive cells compared with DMSO treated controls (percentage of growth inhibition). Combination effects were calculated with the Combenefit software [42] that enables the visualization, analysis and quantification of drug combination effects in terms of synergy and/or antagonism.

Intracellular pH assessment was performed, as follow: Cells were seeded in 96-well black plates following the same 6 × 6 matrix design as described in the *in vitro* cell viability assay. The day after, we replace the growth medium with HHBS buffer and we use the Intracellular pH Assay kit (ab228552, abcam) to run the pH assay by using 100 μL/well of RatioWorks. We incubate the dye-loading plate in cell incubator for 30 minutes, and then incubate the plate at room temperature for another 30 minutes. Drugs were added, we run the pH assay by monitoring the fluorescence at Ex/Em = 490/535 nm (spectrophotometer Spark, Tecan). We used the Online Four Parameter Logistics Calculator to perform our analysis.

### Flow cytometry cell cycle analysis

A549 cells were seeded in six well plates. The day after, each drug was added in triplicate. Twenty-four hours later, adherent and floating cells were collected after trypsinization. The cells were then washed once with PBS followed by PBS containing 0.5% BSA, before being fixed in cold ethanol (70%). The cells were subsequently incubated in PBS containing 10 μg/ml propidium iodide (PI; Invitrogen Villebon-sur-Yvette, France) and 100 μg/ml RNase A (Invitrogen) overnight at 4°C. The data were acquired with a ZE5 cytometer (Biorad) (a minimum of 20 000 cells per sample were analyzed). The DNA content was quantified by using ModFit LT software (Verity Software House, Topsham, ME).

## SUPPLEMENTARY MATERIALS




