# Supplementary material for: High in vitro and in vivo synergistic activity between mTORC1 and PLK1 inhibition in adenocarcinoma NSCLC
Source: Oncotarget. 2021 Apr 13;12(8):859–72. doi: 10.18632/oncotarget.27930 (PMC8057272; doi:10.18632/oncotarget.27930)
Supplement: Supplementary file 1 [file oncotarget-12-859-s001.pdf]

High *in vitro* and *in vivo* synergistic activity between mTORC1 and PLK1 inhibition in adenocarcinoma NSCLC

SUPPLEMENTARY MATERIALS

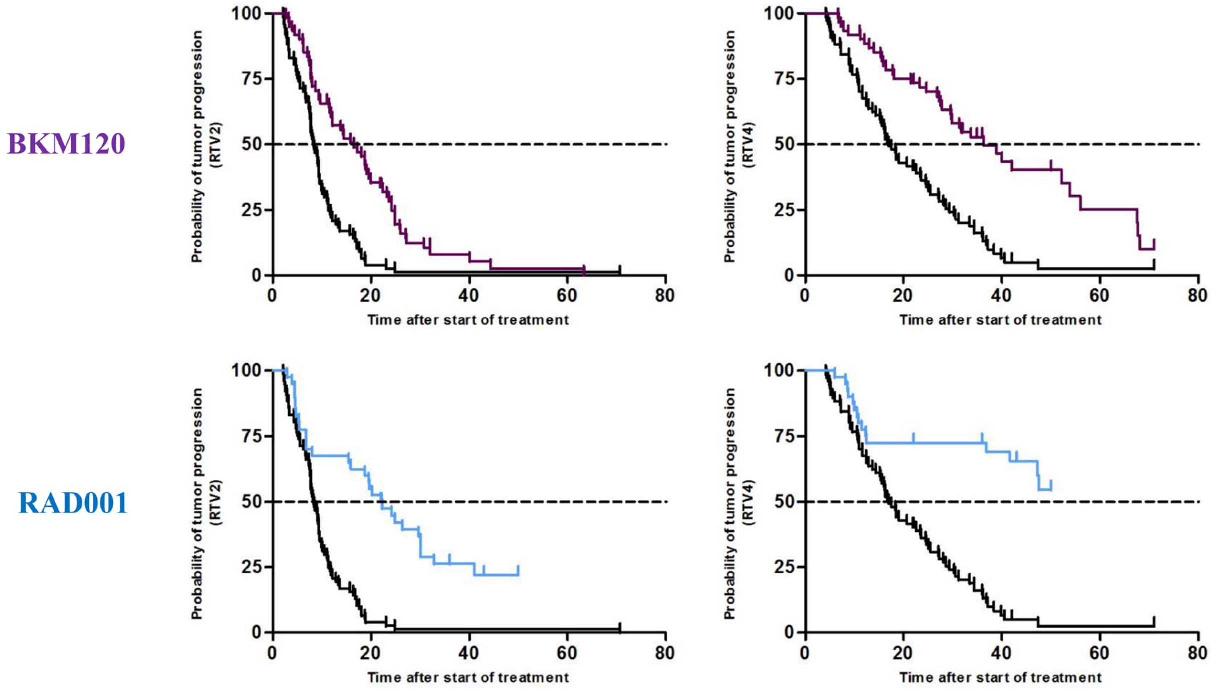

Supplementary Figure 1: *In vivo* efficacy of Pi3K-targeted therapies in NSCLC PDXs. Probability of progression (doubling time and RTVx4) after BKM120 (purple line) and RAD001 (blue line) administration in all treated PDXs. The black lines correspond to the control groups.

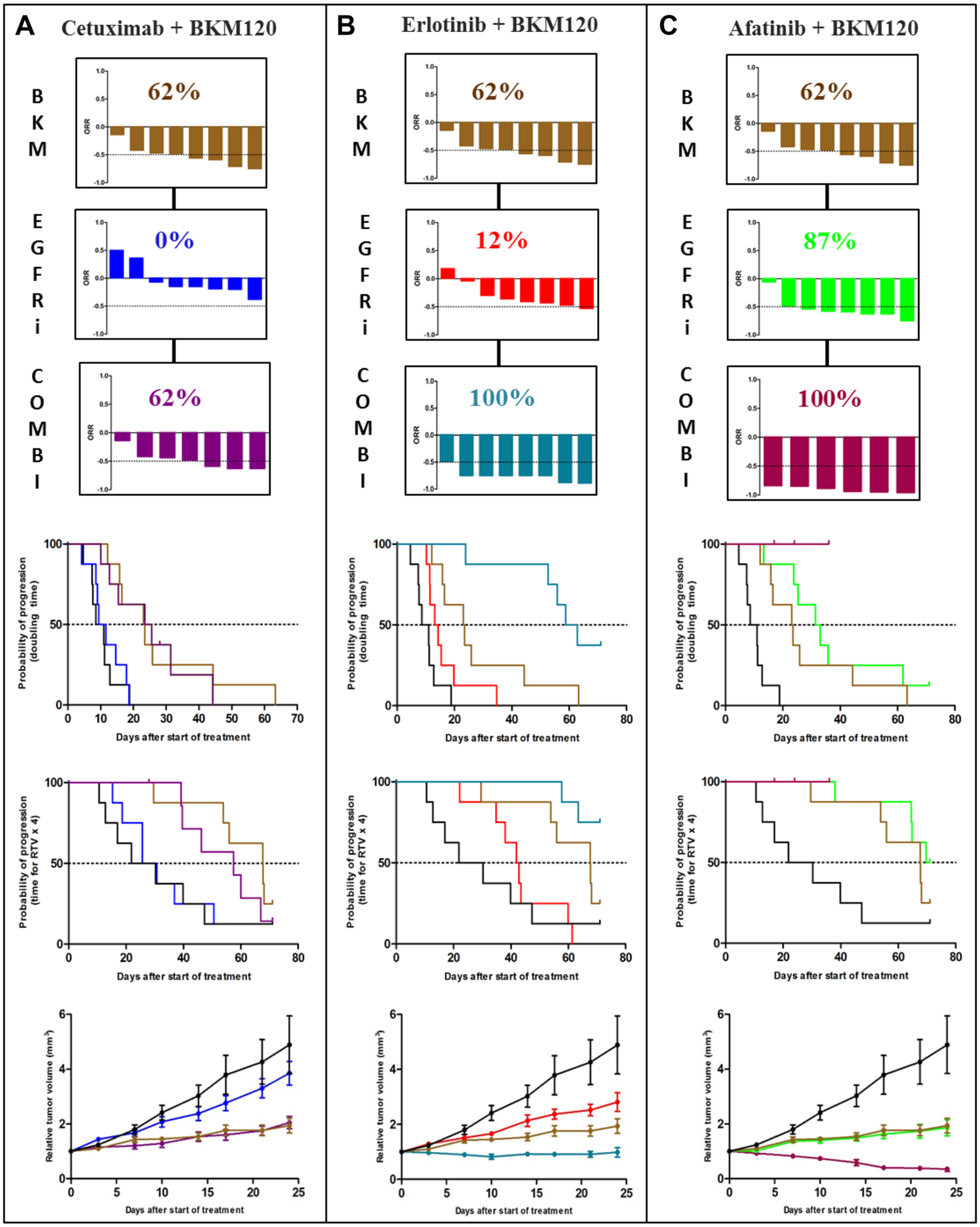

Supplementary Figure 2: *In vivo* efficacy of EGFR-targeted therapies + BKM120 in an *EGFR*- and *PI3KCA*-mutated NSCLC PDX. (**A**) Cetuximab + BKM120. (**B**) Erlotinib + BKM120. (**C**) Afatinib + BKM120. From top to bottom, overall response rate after BKM120, EGFR-TKi, and combination, respectively, probability of progression (doubling time and RTVx4) and RTV curves.

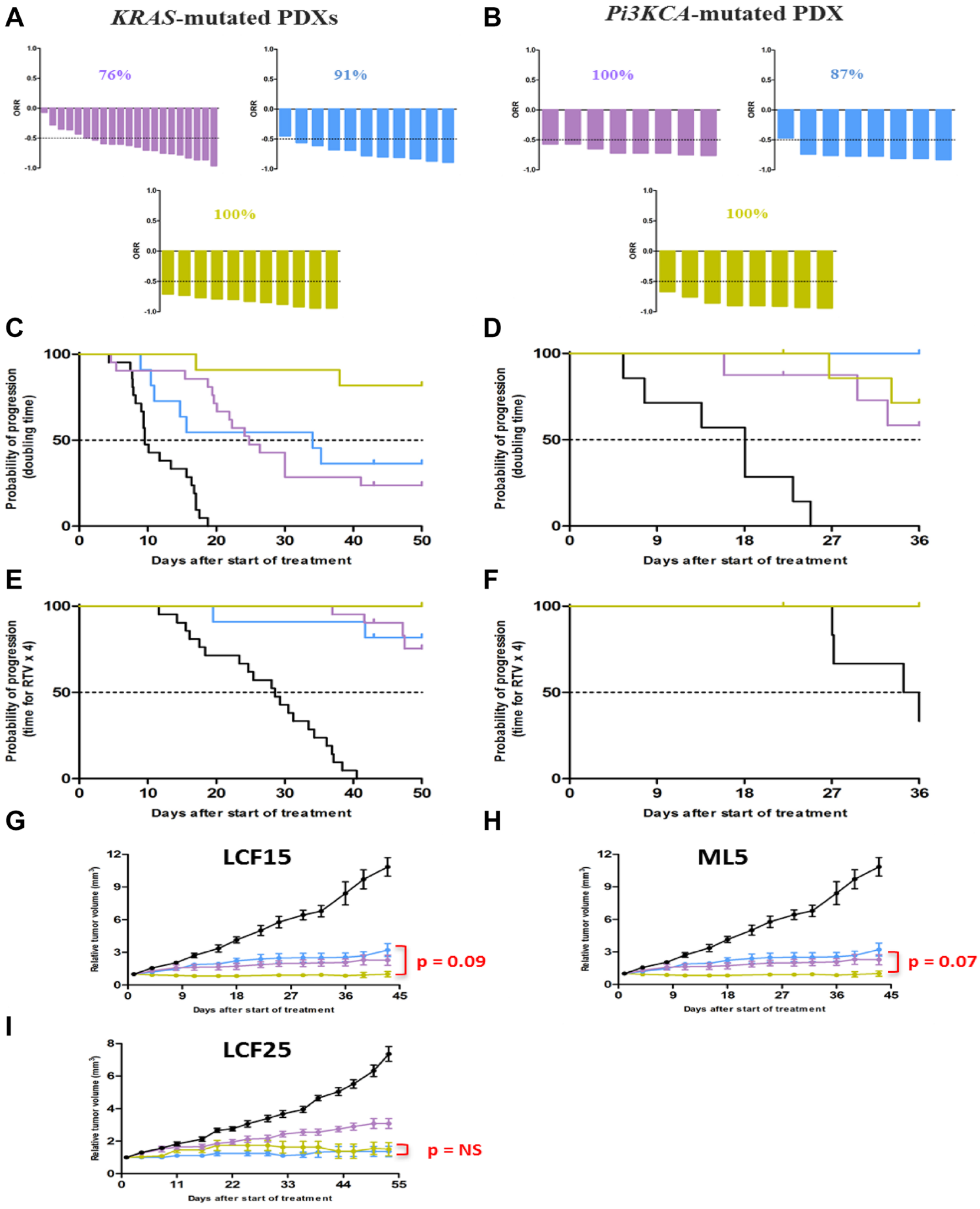

Supplementary Figure 3: *In vivo* efficacy of RAD001 + selumetinib in NSCLC PDX. (**A**) Overall response rate in *KRAS*-mutated PDXs after selumetinib (purple dots), RAD001 (blue dots), and combination (yellow dots). The comparison between the single agents and the combination (Supplementary Figure 3A and 3B) corresponds to the percentage of individual treated mice for which [(RTVV)-1] (defined in the M&M section) was lower to -0.5. (**B**) Overall response rate in *PI3KCA*-mutated PDX after selumetinib (purple dots), RAD001 (blue dots), and combination (yellow dots). Erlotinib + BKM120. C.D.E.F. Probability of progression (doubling time and RTVx4) in KRAS- (**C**, **E**) and PI3KCA-mutated (**D**, **F**) PDXs. (**G**, **H**, **I**) RTV curves of all treated PDXs.

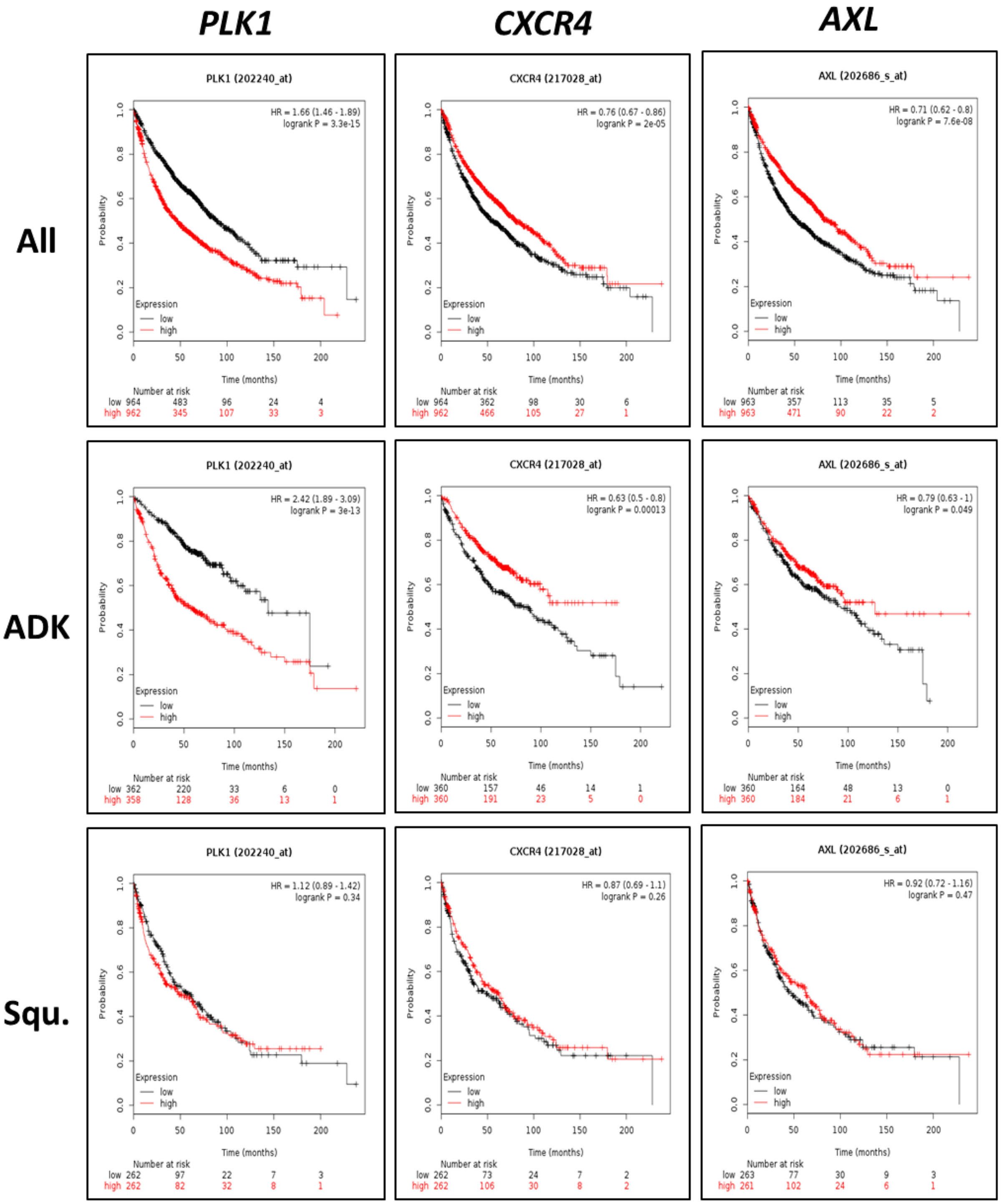

Supplementary Figure 4: Overall survival of NSCLC patients according to the expression of *PLK1*, *CXCR4*, and *AXL* genes (gene chip) in all histological types of NSCLC, adenocarcinoma (ADK) , and squamous cell carcinomas (squ.).

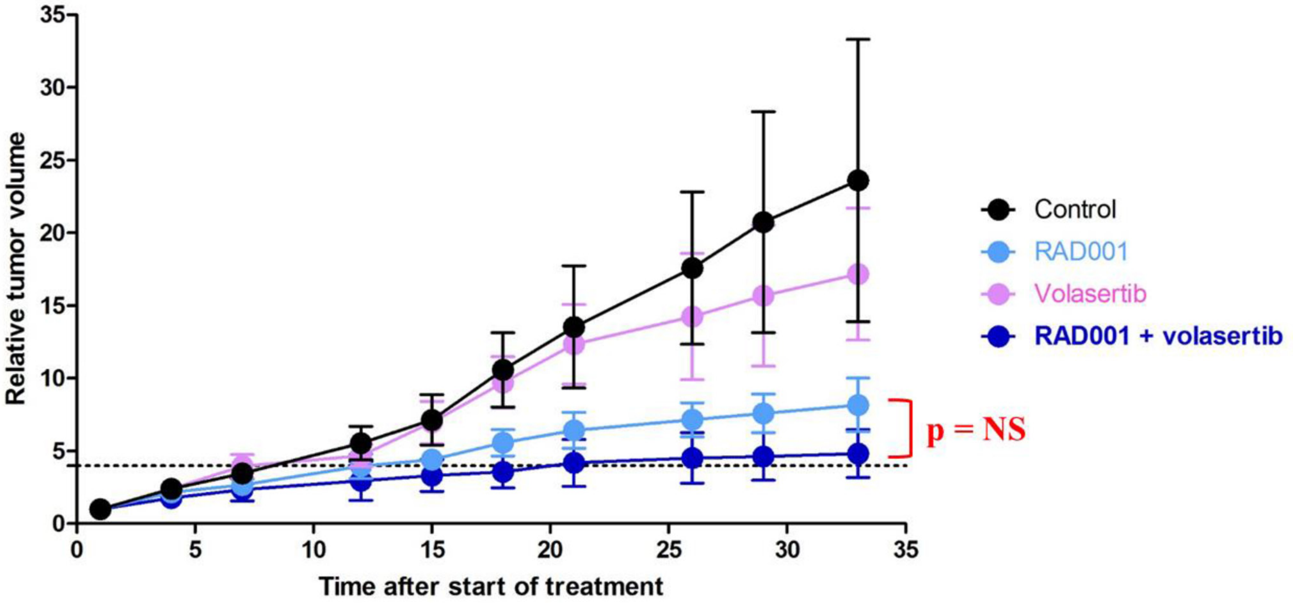

Supplementary Figure 5: *In vivo* efficacy of RAD001 + volasertib in the SC131 squamous NSCLC PDX (Relative Tumor Volume).

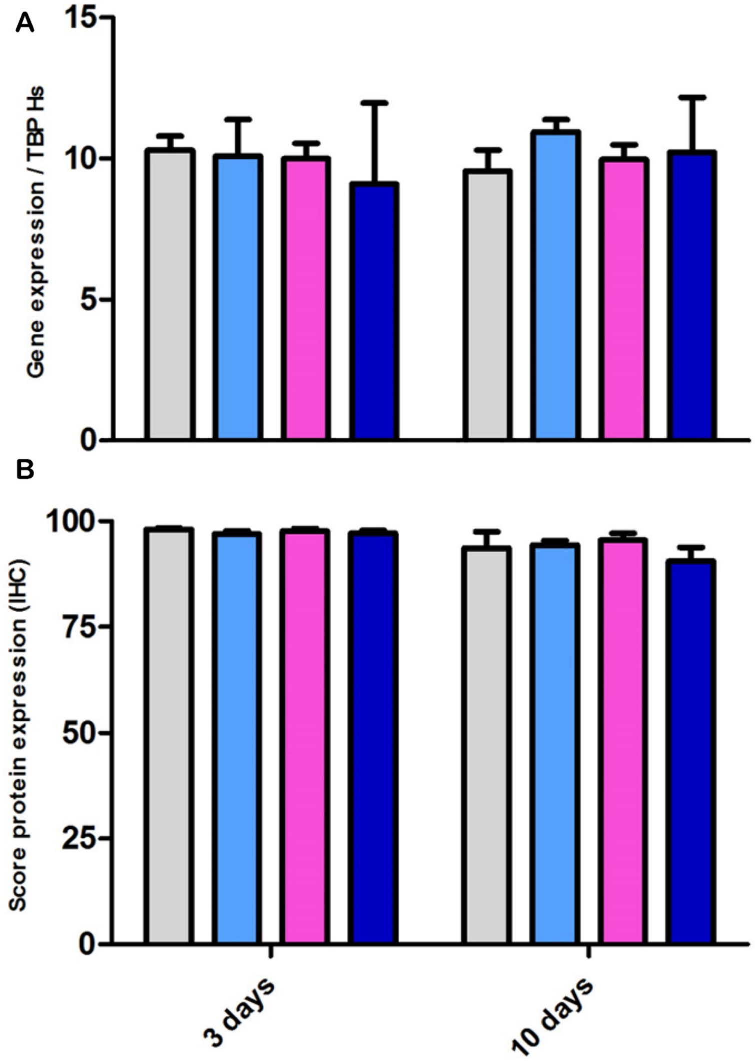

Supplementary Figure 6: PD study in ML1 PDX and Ki67 expression. (**A**) RT-PCR study of Ki67 gene expression. (**B**) IHC study of Ki67 protein expression.

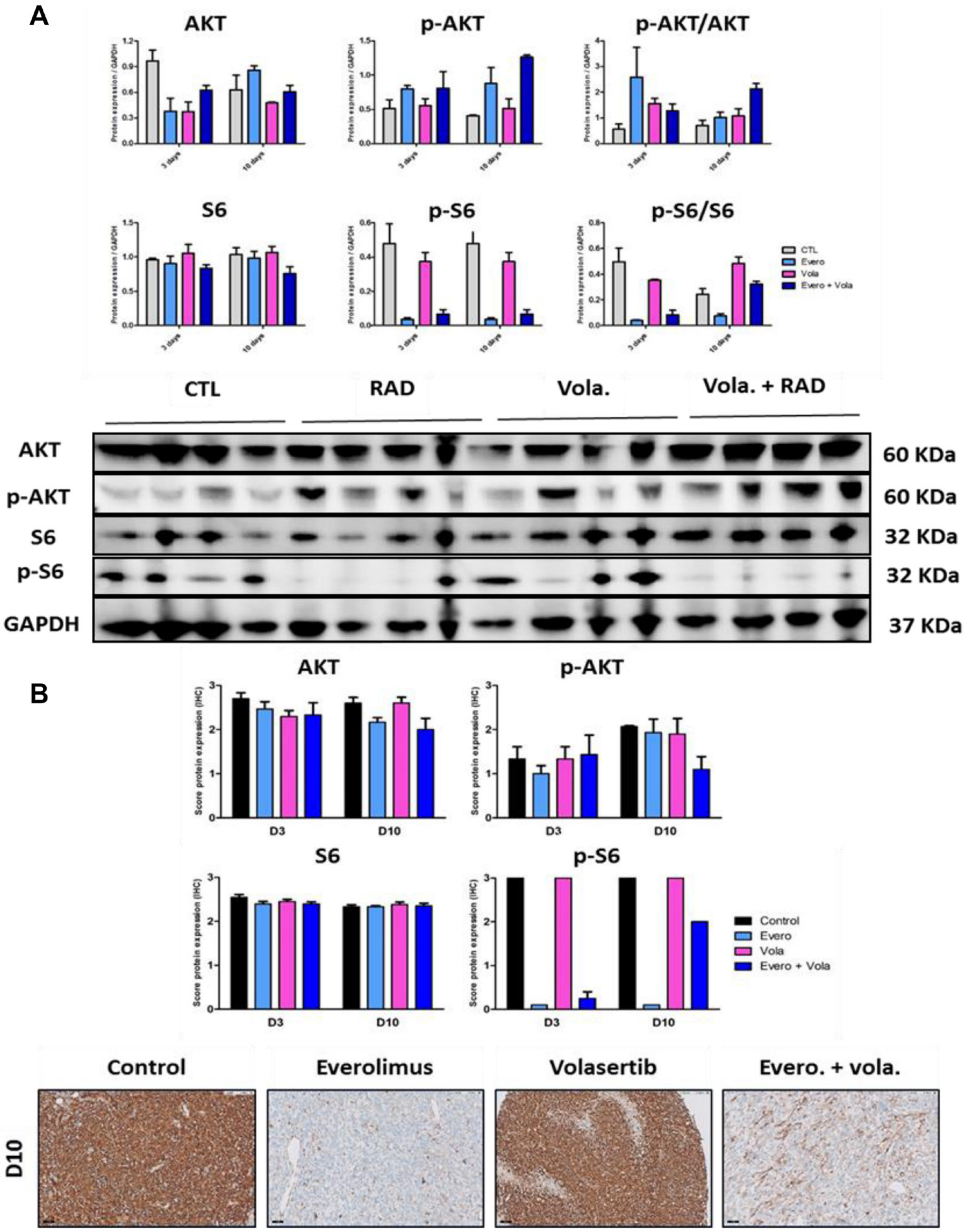

Supplementary Figure 7: PD study in ML1 PDX and Pi3K-related protein expression. (**A**) Western Blot study of Pi3K-related protein expression. (**B**) IHC study of Pi3K-related protein expression.

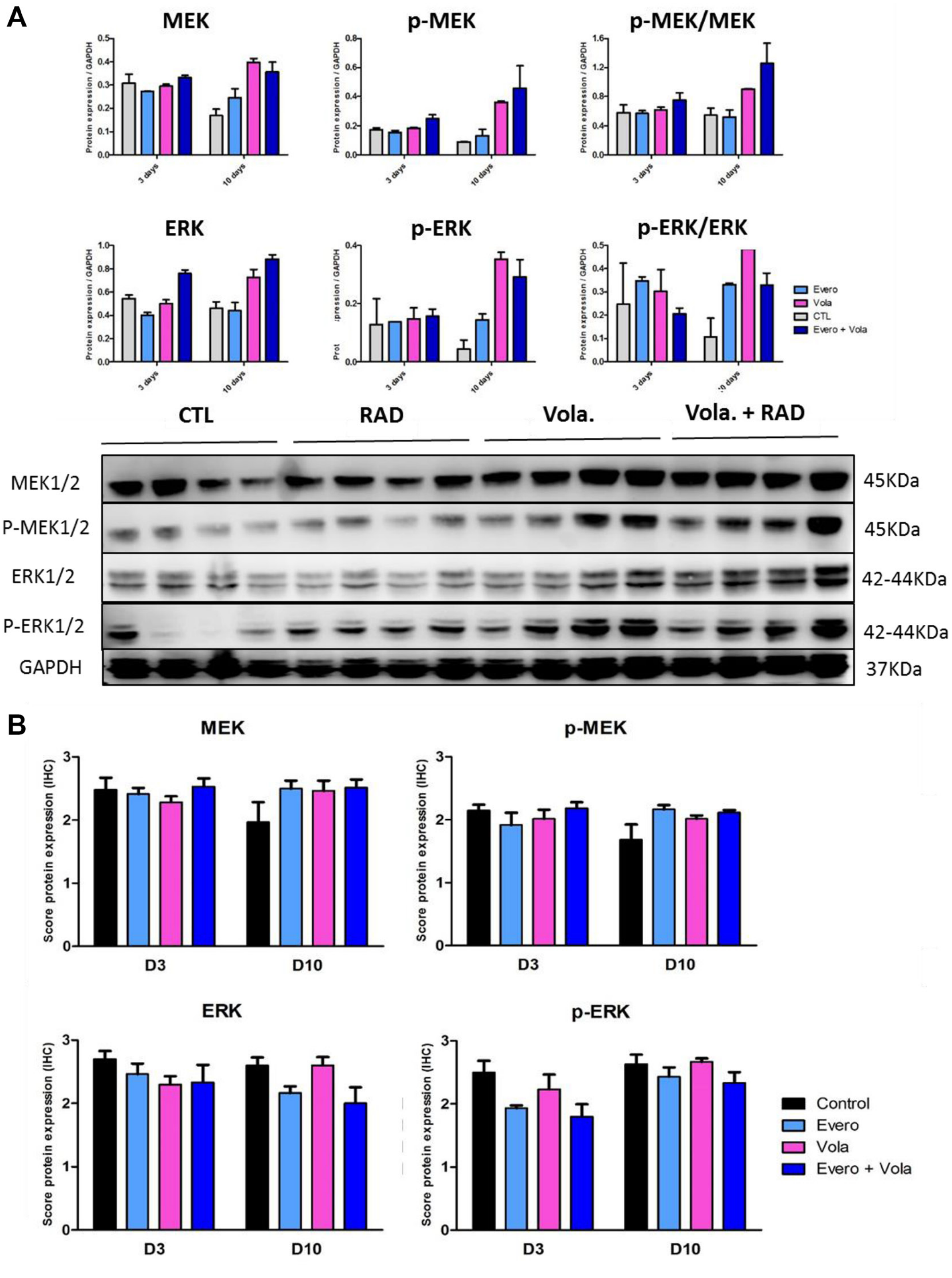

Supplementary Figure 8: PD study in ML1 PDX and MAPK-related protein expression. (**A**) Western Blot study of MAPK-related protein expression. (**B**) IHC study of MAPK-related protein expression.

Supplementary Table 1: NSCLC PDXs and *in vivo* treatments

**NSCLC PDXs**			**LCF4**	**LCF9**	**LCF12**	**LCF15**	**LCF25**	**LCF29**	**ML1**	**ML5**
	Histology	ADK	X	X	X	X	X	X		X
		Large cells							X	
**Tumors**	Mutations	*EGFR*		X			X			
		KRAS				X	X			
		Pi3KCA			X				X	X
	RAD001	mTORC1				X	X	X	X	X
**Mono.**	BKM120	Pi3K	X	X	X	X	X	X	X	X
	Selumetinib	MEK1/2								X
**Combi.**	RAD001 + selumetinib	mTORC1 + MEK1/2				X	X			X
	BKM + EGFR.TKi	Pi3K + EGFR			X					

Abbreviations: ADK, adenocarcinoma; mono., monotherapies; combi., combinations of treatments.

Supplementary Table 2: Predictive factors of response to Pi3K signaling pathway targeting

Groups of genes	Genes	BKM120	RAD001
**HGF**	HGF-Hs	NS	NS
	HGF-Mm	NS	NS
**Membranous Tyrosine Kinase Receptors**	c-MET	NS	0.03
	EGFR	NS	NS
	Her2	NS	0.02
	Her3	NS	NS
	IGF1R	NS	NS
**Proliferation**	MKI67	NS	0.0006
	**PLK1**	NS	**0.004**
**Autophagy**	BECN1	NS	0.05
	LC3B	NS	NS
**Invasion**	**CXCR4**	NS	**0.001**
	CXCL12	NS	0.006
**MAPK**	ELK1	NS	0.004
	ELK3	NS	0.0005
	EGR1	NS	NS
**Hippo**	CTGF	NS	NS
	BIRC5	NS	0.02
	**AXL**	NS	**< 0.0001**
	AREG	0.0002	NS
	RASSF1	0.04	NS
**Pi3K**	EPAS1	NS	0.01
	HIF1A	NS	0.01
	SLC2A1 (GLUT1)	NS	NS
	HMOX1	NS	NS
**Apoptosis**	BCL2L1V1 (BCL-XL)	NS	NS
	MCL1	NS	0.0007
	BCL2	NS	NS
	BCL2L1V2 (BCL-XS)	NS	NS
	BAX	NS	NS
	BAK1	NS	0.02
	BCL2L11 (BIM)	NS	0.04
	BBC3 (PUMA)	NS	0.05
	PMAIP1 (NOXA)	NS	NS
**EMT**	VIM	NS	0.0002
	CDH1	NS	NS
	TWIST1	NS	NS
	SNAI2	NS	0.0002
	SNAI1	NS	0.0006
**Stem cells**	TERT	NS	< 0.0001
	TERC	NS	NS
	PROM1	NS	0.007
	ALDH1A1	0.0001	0.01
	ALDH1A3	NS	NS

Supplementary Table 3: NSCLC PDXs and *in vivo* treatments

NSCLC PDXs			LCF26	LCF29	ML1	ML5	SC131
**Tumors**	Histology	ADK	X	X		X	
		Large cells			X		
		Squamous cell					X
	Main mutations	*EGFR*		X			
		KRAS					X
		Pi3KCA			X	X	
**Mono.**	RAD001	mTORC1	X	X	X	X	X
	Volasertib	PLK1	X	X	X	X	X
**Combi.**	RAD001 + volasertib	mTORC1 + PLK1	X	X	X	X	X

Abbreviations: ADK, adenocarcinoma; mono., monotherapies; combi., combinations of treatments.

Supplementary Table 4: Flow cytometric cell-cycle analyses of A549 cell line

	% G1 phase	% G2 phase	% S phase
**Control**	56	15	29
**RAD001**	70	14	16
**Volasertib**	3	88	9
**RAD001 + Volasertib**	71	14	15

Supplementary Table 5: List of the proteins studied by Western Blot

ANTIBODY	SPECIES	SUPPLIER	REFERENCE
Phospho-S6 Ribosomal Protein (Ser235/236)	R	CST	2211
Akt	R	CST	9272
Phospho-Akt (ser 473)	R	CST	4058
Phospho-MEK1/2 (Ser217/221)	R	CST	9154
MEK1/2	R	CST	9122S
Phospho-p44/42 MAPK (Thr202/Tyr204)	R	CST	4377 (197G2)
p44/42 MAPK	R	CST	9102
S6 Ribosomal Protein (5G10)	R	CST	2217
PLK1	R	CST	4513
Ca9 (D47G3)	R	CST	5649
GAPDH	R	CST	2118

Supplementary Table 6: Densitometry readings/intensity ratio of each band of Western Blot analyses. See Supplementary Table 6

Supplementary Table 7: List of the proteins studied by IHC

ANTIBODY	Product	Clone	Device	pH	Incubation	Dilution	Amplification
S6	Abcam Ab40820	Rabbit polyclonal	DAKO Autostainer	pH6 10 mn	60 min	1/300	no
PLK1	Cell Signaling #4513	Rabbit monoclonal clone : 208G4	BOND RX	pH9 10 min	60 min	1/75	no
CD34	DAKO M716501	Mouse monoclonal clone : QBend10	BOND RX	pH9 10 min	30 min	1/200	no
Akt	Santa Cruz sc-8312	Rabbit polyclonal	DAKO Autostainer	ph6 10 mn	60 min	1/100	no
P-Akt	Cell Signaling #3787	Rabbit monoclonal clone : 736E11	BOND RX	pH9 20 min	over-night 4°C	1/50	LINKER + ABC
ERK	Abcam Ab32537	Rabbit monoclonal clone : Y72	DAKO Autostainer	pH6 10 mn	60 min	1/200	no
P-ERK	Abcam Ab194770	Rabbit polyclonal	DAKO Autostainer	pH9 10 mn	60 min	1/250	no
MEK	Abcam Ab32091	Rabbit monoclonal clone : E342	DAKO Autostainer	pH9 10 mn	60 min	1/75	no
P-MEK	Abcam Ab96379	Rabbit monoclonal clone : EPR3338	DAKO Autostainer	pH9 10 mn	60 min	1/600	no
