# Supplementary material for: High in vitro and in vivo synergistic activity between mTORC1 and PLK1 inhibition in adenocarcinoma NSCLC
Source: Oncotarget. 2021 Apr 13;12(8):859–72. doi: 10.18632/oncotarget.27930 (PMC8057272; doi:10.18632/oncotarget.27930)
Supplement: Supplementary file 2 [file oncotarget-12-859-s002.docx]

**Supplementary Table 6: Densitometry readings/intensity ratio of each band of Western Blot analyses**

| **Model** | **Prot.** | **Control** | | | **RAD** | | | **Vola.** | | | **RAD + Vola.** | | |
| --- | --- | --- | --- | --- | --- | --- | --- | --- | --- | --- | --- | --- | --- |
|  |  | **Protein** | **GAPDH** | **Ratio** | **Protein** | **GAPDH** | **Ratio** | **Protein** | **GAPDH** | **Ratio** | **Protein** | **GAPDH** | **Ratio** |
| **ML1** | **PLK1** | 512499 | 57580982 | 0,009 | 40730,66 | 49216519 | 0,001 | 585394,9 | 44803146 | 0,013 | 469752,6 | 67029504 | 0,007 |
|  |  | 468418,3 | 51820871 | 0,009 | 83610,15 | 39209124 | 0,002 | 301353,3 | 52651647 | 0,006 | 167843,2 | 42877975 | 0,004 |
|  |  | 248890,6 | 44035696 | 0,006 | 51889,05 | 45178937 | 0,001 | 201657,9 | 56327171 | 0,004 | 36477,58 | 56545143 | 0,001 |
|  | **CAIX** | 235500,5 | 3190630 | 0,074 | 669,35 | 2823812 | 0,000 | 101440,1 | 2613886 | 0,039 | 130378,6 | 3116290 | 0,042 |
|  |  | 283316,1 | 3044920 | 0,093 | 65753,11 | 3124003 | 0,021 | 107535,7 | 3007879 | 0,036 | 135262,8 | 3434944 | 0,039 |
|  |  | 191342,1 | 3118390 | 0,061 | 276458,7 | 3053689 | 0,091 | 69085,46 | 2731536 | 0,025 | 162436,9 | 3251376 | 0,050 |
|  |  | 130471,4 | 2803099 | 0,047 | 156041,6 | 3093682 | 0,050 | 120227,4 | 2816127 | 0,043 | 115543,3 | 3324684 | 0,035 |
| **A549** | **PLK1** | 116929598 | 117330435 | 0,997 | 28415725 | 59262020 | 0,479 | 11191029 | 80077964 | 0,140 | 40117533 | 55029847 | 0,729 |
|  | **CAIX** | 23749468 |  | 0,202 | 10782334 |  | 0,182 | 9263026 |  | 0,116 | 2597665 |  | 0,047 |
|  | **HIF1** | 2915423 |  | 0,049 | 976342 |  | 0,012 | 2002495 |  | 0,036 | 1623018 |  | 0,029 |
|  | **AKT** | 48339527 |  | 0,878 | 26108703 |  | 0,441 | 14790897 |  | 0,185 | 23143949 |  | 0,421 |
|  | **p.AKT** | 25809801 |  | 0,220 | 32382718 |  | 0,404 | 2630803 |  | 0,033 | 36710279 |  | 0,667 |
|  | **S6** | 162855422 |  | 2,748 | 52235907 |  | 0,949 | 56991226 |  | 0,712 | 48204369 |  | 0,876 |
|  | **p.S6** | 133632635 |  | 1,669 | 2781160 |  | 0,047 | 104996721 |  | 1,311 | 1621694 |  | 0,029 |
|  | **MEK** | 111397541 |  | 2,024 | 50754958 |  | 0,634 | 65019291 |  | 0,812 | 37419655 |  | 0,680 |
|  | **p.MEK** | 39093467 |  | 0,333 | 29985617 |  | 0,545 | 59820306 |  | 0,747 | 22604650 |  | 0,411 |
|  | **ERK** | 69881956 |  | 1,179 | 30269325 |  | 0,511 | 22418907 |  | 0,280 | 19167388 |  | 0,348 |
|  | **p.ERK** | 18530723 |  | 0,231 | 20442472 |  | 0,255 | 70433082 |  | 0,880 | 23493547 |  | 0,427 |

**Abbreviations:** Prot., WB-studied proteins; Ratio, ratio protein/GAPDH.
